# Anti-erosion effect of an experimental varnish on eroded dentin

**DOI:** 10.1590/0103-6440202305325

**Published:** 2023-07-17

**Authors:** George Monteiro, Antonia Patricia Oliveira Barros, Gabriela Carvalho Santos Fernandes, Fernanda Ferreira de Albuquerque Jassé, Milton Carlos Kuga, Cristiane de Melo Alencar

**Affiliations:** 1 Department of Restorative Dentistry, Federal University of Pará, Belém, Pará, Brazil; 2 Department of Restorative Dentistry, Sao Paulo State University, Araraquara, São Paulo, Brazil; 3 Department of Restorative Dentistry, School of Dentistry, University Center of the State of Pará, Belém, Pará, Brazil

**Keywords:** dentin, erosion, nano-hydroxyapatite, profilometry

## Abstract

This in vitro study evaluated the effect of an experimental varnish containing 20% nano-hydroxyapatite (nHAP) associated with 5% stannous chloride (SnCl_2_) against erosive-abrasive wear on bovine dentin. Samples of bovine cervical dentin were pre-eroded (0.3% citric acid, pH 2.6 for 10 minutes) and randomized into 4 groups (n=10): Control group - experimental varnish without active ingredient (CG); experimental varnish containing 20% nHAP (nHG); experimental varnish containing 5% SnCl_2_ (24.800 ppm Sn^2+^) (SnG); experimental varnish containing 20% nHAP associated with 5% SnCl_2_ (18.300 ppm Sn^2+^) (nHSnG). After applying the materials, the erosive-abrasive challenges were performed for five days. Erosive dentin loss and analysis of the pattern of dentinal obliteration were performed by 3D confocal laser microscopy. A one-way ANOVA/Bonferroni test was performed to analyze the data (α=0.05). The SnG and nHSnG experimental groups presented more effectiveness in preventing erosive wear when compared to the other groups (p<0.05). There was no statistically significant difference between the SnG and nHSnG groups (p = 0.731) in tooth structure dentin loss. Regarding the amount of open dentinal tubules, the highest amount of obstructed dentinal tubules was demonstrated in SnG and nHSnG (p < 0.05) when compared to the others. Between SnG and nHSnG there was no significant difference (p = 0.952) in the amount of closed dentinal tubules in the dentin. Experimental varnishes containing 5% SnCl_2_ associated or not with 20% nHAP showed to be a promising strategy in preventing erosive-abrasive wear of dentin. In addition, nHSnG was able to obliterate dentinal tubules.

## Resumo

Este estudo in vitro avaliou o efeito de um verniz experimental contendo 20% de nano-hidroxiapatita (nHAP) associado a 5% de cloreto estanoso (SnCl_2_) contra o desgaste erosivo-abrasivo da dentina bovina. As amostras de dentina cervical bovina foram pré-erodificadas (0,3% de ácido cítrico, pH 2,6 durante 10 minutos) e aleatorizadas em 4 grupos (n=10): Grupo controle - verniz experimental sem ingrediente ativo (GC); verniz experimental contendo 20% nHAP (GnH); verniz experimental contendo 5% SnCl_2_ (24.800 ppm Sn^2+^) (GSn); verniz experimental contendo 20% nHAP associado a 5% SnCl_2_ (18.300 ppm Sn^2+^) (GnHSn). Após a aplicação dos materiais, os desafios erosivo-abrasivos foram realizados durante cinco dias. Perda de dentina erosiva e análise do padrão de obliteração dentinária foram realizadas por microscopia laser confocal 3D. Foi realizado o teste ANOVA/Bonferroni unidireccional para analisar os dados (α=0,05). Os grupos experimentais GSn e GnHSn apresentaram maior eficácia na prevenção do desgaste erosivo quando comparados com os outros grupos (p<0,05). Não houve diferença estatisticamente significativa entre os grupos GSn e GnHSn (p = 0,731) na perda de dentina da estrutura dentária. Relativamente à quantidade de túbulos dentinários abertos, a maior quantidade de túbulos dentinários obstruídos foi demonstrada em GSn e GnHSn (p < 0,05) quando comparada com os outros grupos. Entre GSn e GnHSn, não houve diferença significativa (p = 0,952) na quantidade de túbulos dentinários fechados na dentina. Os vernizes experimentais contendo 5% de SnCl2 associados ou não a 20% de nHAP mostraram ser uma estratégia promissora na prevenção do desgaste erosivo-abrasivo da dentina. Além disso, o GnHSn conseguiu obliterar os túbulos dentinários.

## Introduction

Tooth wear is a clinical condition defined as the result of physical or chemophysical processes (dental erosion, attrition, abrasion) that generate the accumulative surface loss of mineralized tooth substance ^1^. Tooth wear is known to be an increasingly complex challenge in dentistry due to the significant increase in its incidence ^2^. Although some degree of tooth wear is common throughout life, it can be considered excessive when the pattern of surface loss is disproportionate to the individual's age ^3^. Although the most recommended way to prevent tooth wear is continuous fluoride application ^4^, this measure alone does not seem to be enough to reduce and resist erosive challenges ^5^.

Recently available evidence addresses different strategies for preventing and controlling erosive wear. These include new bioactive polymers, fillers, or toothpastes that assist in calcium-phosphate remineralization ^6^ ^,^ ^7^. In addition, the use of fluorides ^8^, high-power lasers ^9^, sealants ^10^, and, for cases with large tissue loss, adhesive restorations ^11^ have also been reported. Since hydroxyapatite is the main constituent of the inorganic phase of both enamel and dentin, products with similar chemical and structural characteristics, capable of providing calcium and phosphate ions in adequate concentrations, are also alternatives to restructuring dentin ^12^. In the context of erosion, its application reflects promising results through the replacement of calcium and phosphate ions on demineralized surfaces and the formation of a protective film on the tooth surface, with cohesive and acid resistance ^13^. In addition, they act by occluding the dentinal tubules, making it difficult for external stimuli to access the pulp ^14^.

The mechanism of action of 5% stannous chloride is still unclear. However, it is known that isolated tin has great potential to form a protective barrier ^15^. This barrier forms from the surface precipitation of the ion when the organic matrix is removed, reacting with the underlying mineralized tissue, and forming different salts ^16^. In addition, tin can directly inhibit dentin matrix metalloproteinases (MMPs), which are collagen-degrading enzymes ^17^. It is not known for sure how the inhibition occurs, but it is hypothesized that tin interacts with MMPs by blocking binding sites and inhibiting their activity, resulting in greater protection of dentin from dental erosion ^18^. 

To date, only one study has been published addressing the action of a varnish containing 5% stannous chloride in controlling dentin erosion ^19^ and, according to the knowledge of the authors, none evaluated the nano-hydroxyapatite associated with this component, which would be an innovative alternative for future treatments of this condition. Furthermore, the effects of stannous chloride at a concentration of 5% against erosion and abrasion in the dentin are still uncertain in the literature, which justifies its choice in this study. Thus, this study aimed to evaluate the effectiveness of an experimental varnish containing nano-hydroxyapatite (nHAP) associated with 5% stannous chloride (SnCl_2_) against erosion and abrasion in bovine dentin. The null hypotheses tested were: H01: There is no difference in the loss of tooth structure between the experimental groups tested; H02: There is no difference in the pattern of obliteration between the groups evaluated after anti-erosion treatment. 

## Materials and methods

### Sample preparation

Bovine cervical dentin blocks (60 samples) were obtained from 60 healthy bovine incisors using a water-cooled double-sided diamond disc (Buehler, Lake Bluff, Illinois, United States). The root dentin blocks (4×4×2mm^3^) were cut using an Isomet cutting machine (Buehler, Lake Bluff, Illinois, United States) with a double-sided diamond disc (Extec, Enfield, Connecticut, United States). Then, the blocks were hand polished in a circular motion using #600 and #1200 silicon carbide sandpapers (3M, Sumaré, São Paulo, Brazil). After polishing, the samples were immersed in an ultrasonic bath (Euronda Spa, Montecchio Precalcino, Vicenza, Italy) with distilled water (Milli-Q, Merck Millipore Corporation, Darmstadt, Germany) for 5 min and then kept in a humid environment (Milli-Q water), at 4 °C until the time of the experiment.

### Specimen selection

The 60-dentin blocks were subjected to a base surface microhardness test (BSM). BSM was performed using Knoop microhardness (Surftest Mitsutoyo South American, São Paulo, Brazil) under a load of 50g for 5 s ^20^. Five indentations 100 µm apart were performed in the central area of the dentin surface. After the test, the evaluation of data normality (Shapiro-Wilk test) was performed using SPSS software version 13.0 (SPSS, Tulsa, Oklahoma, United States). Twenty dentin blocks were excluded as they had anomalous microhardness values and 40 dentin samples were numbered and randomized into four groups (n=10). Therefore, mean baseline microhardness values were not statistically different between groups (analysis of variance [ANOVA]; α = 0.05).

### Initial erosion

An initial erosive lesion was created by applying 0.3% citric acid (pH 2.6), for 10 minutes ^19^. This protocol was performed in 24-well acrylic plates, and each sample was inserted into a specific well. Then, each sample was washed with distilled water for 10 seconds using a millimeter pipette and dried with absorbent paper. Half of the eroded surface of the samples was covered with unplasticized polyvinyl chloride (UPVC) tape to leave a 4 × 2 mm exposure window uncovered ^21^.

### Varnish treatment and erosive-abrasive challenge

After initial erosion and protection of half of the specimen surface with UPVC tape, the varnishes were applied in the respective groups (n = 10): Control group - experimental varnish without active ingredient (CG); experimental varnish containing nHAP (nHG); experimental varnish containing 5% SnCl_2_ (24.800 ppm Sn^2+^) (SnG); experimental varnish containing nHAP associated with 5% SnCl_2_ (18.300 ppm Sn^2+^) (nHSnG). The basic composition of experimental varnishes includes film-forming polymer (ethylcellulose), artificial resin (colophony), solvent (ethanol), essence (saccharin), and demineralized water (Faculty of Pharmacy at USP, São Paulo, Brazil). The SnCl_2_ varnish was prepared by dissolving SnCl_2_ (ca. 5%) and resin products in ethanol 96%. A homogeneous solution was obtained by slowly adding the solids to ethanol with vigorous stirring. Then synthetic nano-hydroxyapatite powder (Sigma-Aldrich) was added a density of 2 to 6g/cm^3^, a surface area of 10 to 15 m^2^/g, and a particle diameter < 150nm. Next, the viscous solution (20% nano-hydroxyapatite and 5% SnCl_2_) was placed in plastic containers (n45), protected from light exposure, and maintained in an aging process at 65 °C and 30% RH (relative humidity) as described earlier ^22^. To determine the Sn concentrations, a specific electrode for fluorine ion (9609 BN - Orion) and an ion analyzer (Orion 720 A^+^) were used, previously calibrated with 5 standards: 2.0; 4.0; 8.0 and 16.0 and 32 µg Sn/mL. The 20% nano-hydroxyapatite concentration was used based on the study by Souza, et al. ^23^, and 5% SnCl_2_ is an adaptation of Alencar, et al. ^19^. 

The pH of all varnishes was measured with indicator paper (± 0.5 units): Experimental varnish without active ingredient (pH=6.81); experimental varnish containing nHAP (pH=7.03); experimental varnish containing 5% SnCl_2_ (pH=6.69); experimental varnish containing nHAP associated with 5% SnCl2 (pH=7.43). The experimental materials showed similar color and consistency.

The varnishes were applied in a thin layer with a disposable brush and the specimens were stored in artificial saliva for 6 h. Subsequently, the varnishes were removed with acetone solution (1:1) and cotton swabs, avoiding contact with the dentin surface ^24^. After finishing the treatments, the samples were submitted to acid cycling for five days. The specimens from each group were immersed in 0.3% citric acid solution (pH = 2.6 for 10 min) and then immersed in artificial saliva (Concentration of components in 0.96 g/1000 mL - KCl; NaCl; MgCl_2_; K_2_HPO_4_; CaCl_2_; Carboxymethylcellulose; Sorbitol 70%; Nipagin; Nipazole and deionized water) for 60 minutes. The samples were embedded in acrylic resin and positioned in a brushing machine (MEV-2T Odeme, Joaçaba, Santa Catarina, Brazil) for simulated brushing twice a day, calibrated in 45 cycles of 150 g for 15 s. Simulated brushing was performed 30 min after the 1st and 4th acid challenge on each day of the cycle. The entire protocol was performed at an average temperature of 25°C and, at the end of each day; the samples were stored at 100% humidity ^6^.

### Tooth structure loss (TSL)

The 100% humidity of the specimens was maintained throughout the experiment. The surface topography of the samples was measured by a 3D confocal laser microscope (LEXT OLS4000, Olympus, Tokyo, Japan). The capture was performed using a chromatic confocal sensor with an axial source of white light, a scan speed of 2 m/s, and a refractive index of 10.000. An area of 1 mm × 1 mm was obtained from the center of each sample within the 4 × 2 mm exposure window. The analysis determined the tooth structure loss (TSL), defined as the height difference (Δ height) between the untreated surface (baseline) and the treated and challenged surface. Values in µm were calculated using Nanovea Professional 3D software and this methodology was performed according to Alexandria, et al. ^25^.

### Obliteration Pattern - Tubule Count

Images were obtained with a 3D confocal laser microscope (LEXT OLS4000), at 20 kV. Four distinct fields were initially evaluated, and from the one most representative of the specimen, a fixed image was obtained at the same location for all samples at a magnification of 500×, to evaluate the precipitation of residues on the dentin surface. From this location, another image with 1000× magnification was obtained to count open and unobstructed dentinal tubules. A single operator obtained the image. Two calibrated and independent examiners classified the presence of residues as described by Kuga, et al. ^26^. Two other examiners counted the open dentinal tubules for each specimen image. Intra-examiner agreement analysis was considered with coefficient Kappa=1.0. The average obtained between both was determined for the specimen under analysis.

### Statistical analysis

SPSS software version 13.0 (SPSS) was used to perform the statistical analysis. The evaluation of the parametric distribution of the data was performed using the Shapiro-Wilk test and homoscedasticity was also verified. Two-way ANOVA followed by Tukey's test was used to analyze erosive tooth loss. The tubule count data were subjected to Kruskal-Wallis and Dunn test. The level of significance was set at α = 0.05.

## Results

### Tooth structure loss (TSL)

The results are shown in Table 1. SnG and nHSnG experimental groups were more effective in preventing tooth structure loss (TSL) when compared to the other groups (p < 0.05). There was no statistically significant difference between the SnG and nHSnG groups (p = 0.731). The negative control group showed significantly higher TSL when compared to the other groups for both substrates (p < 0.05).

**Table 1:** Mean and standard deviation (SD) of tooth structure dentin loss (TSL) values ​​in µm

Groups	TSL (µm) / Mean (±SD)
CG	-181.63 (±17.82)^a^
nHG	-48.35 (±2.79)^b^
SnG	-16.09 (±0.90)^c^
nHSnG	-15.87 (±1.11)^c^

*Different letters show a statistically significant difference (p<0.05) between groups.

### Obliteration Pattern - Tubule Count

**Figure 1:**
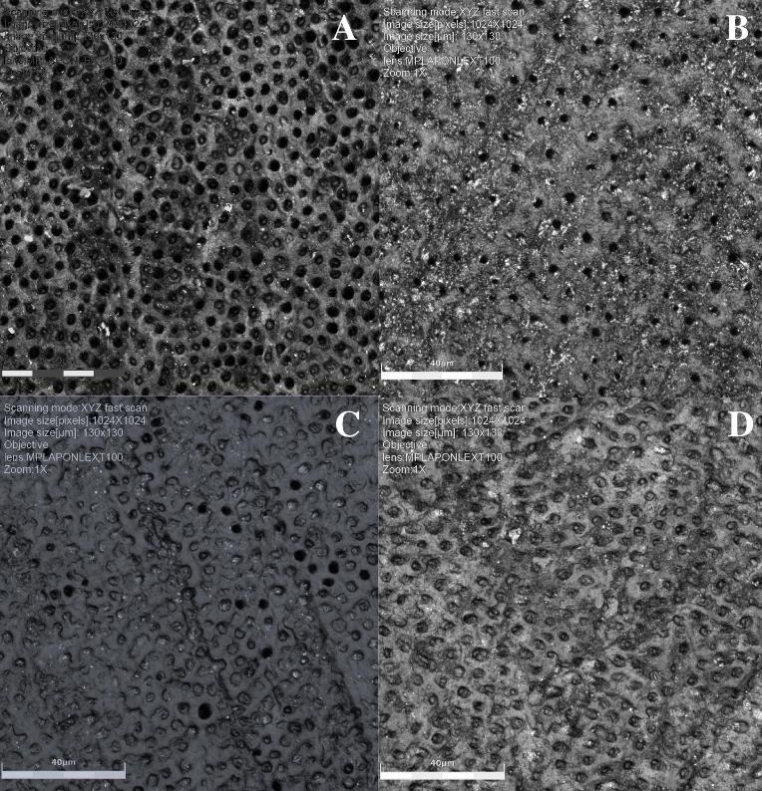
(A) Control group; (B) experimental varnish containing nHAP; (C) experimental varnish containing 5% SnCl_2_ and (D) experimental varnish containing nHAP associated with 5% SnCl_2_.

Regarding the amount of open dentinal tubules, the highest amount of obstructed dentinal tubules was demonstrated in SnG and nHSnG (p < 0.05) when compared to the others. Between SnG and nHSnG there was no significant difference (p = 0.952). Table 2 shows the median, maximum and minimum values, first and third quartiles of the amount of open dentinal tubules on the dentin surface, depending on the groups evaluated. Figure 1 illustrates the representative images of the dentinal surface characteristic after performing the desensitization protocols, and the control group.

**Table 2:** Median, maximum, and minimum values and first (1Q) and third quartile (3Q) of the amount of open dentinal tubules (unit) in the dentin, as a function of the dentinal desensitization protocols used.

Groups	Median	Maximum	Minimum	1Q-3Q
CG	981^a^	1238	456	907.3-1342.7
nHG	43^b^	127	19	47.1-134.2
SnG	17^c^	89	5	17.9-100.3
nHSnG	12^c^	75	4	13.2-83.6

*Different letters show a statistically significant difference (p<0.05) between groups.

## Discussion

In the present study, the erosion-protective effect of experimental varnishes was analyzed under simulated exposure to citric acid at a pH commonly found in erosive beverages ^27^. In contrast to previous studies ^5^, in the present experiment, a single application of experimental varnish was performed after an initial erosive attack, as may occur in a patient suffering from dental erosion. The material was applied only once to analyze its protective effect against erosion/abrasion.

The experimental varnish containing 5% SnCl_2_ reduced structure loss compared to the control, which is consistent with a previous study by Alencar, et al. ^19^. In that study, although SnCl_2_ was associated with NaF, the authors obtained similar results in the prevention of TSL. In addition, 5% SnCl_2_ associated with NaF showed better results in preventing TSL when compared to 5% NaF (Duraphat) ^19^, implying to say that SnCl_2_ enhances the protective effect against erosion/abrasion, corroborating with our results. Furthermore, this varnish also demonstrated a statistically significant pattern of obliteration in the confocal laser microscopy analysis after 5 days of exposure to 0.3% citric acid (pH 2.6) and simulated brushing, in relation to the control. Thus, the null hypotheses H01 and H02 were rejected. Ganss, et al. ^28^ report that in dentin the protection observed with SnCl_2_ may be related to the same mechanisms explained for enamel, showing that an extensive tin-rich deposit forms in dentin after application of the compound, capable of occluding the dentinal tubules. Addy and Mostafa ^29^ explain that its protective effect is the result of the mineral content formed in dentin since tin ion is a potent reagent with hydroxyapatite. This explanation suggests that precipitates formed in dentin after the application of tin-containing material are as resistant to acids as precipitates formed in enamel, acting similarly on dentin and enamel, corroborating with the results obtained by Ganss, et al. ^28^.

However, more research is needed to better understand the mechanisms involved in the protective action of SnCl_2_ on the dentin surface. The present study also demonstrated that experimental varnish containing nHAP obtained statistically significant results in relation to control for both TSL and pattern of obliteration. This is because nHAP is a framework for dentin remineralization processes after erosion ^30^. However, such satisfactory results were not obtained when comparing it with SnCl_2_ varnish. It is known that nano-hydroxyapatite particles (nHAP) stand out for having characteristics closer to biological apatites than the large synthesized HAP particles ^31^. Thus, it is assumed that SnCl_2_, when forming ions of lower molecular weight ^32^ was able to form a more homogeneous layer on the dentin surface and with greater adhesion. Thereby, it reduced both TSL and obliterated a greater number of tubules.

Although the nHAP-containing varnish did not present such satisfactory results when compared to the SnG group, the findings of the present study showed that the association of nHAP with 5% SnCl_2_ presents excellent results both in TSL and in the pattern of tubule obliteration. Besinis et al. ^30^ reported that the infiltration of hydroxyapatite particles into demineralized dentin increased significantly when it was combined with another agent. It can be assumed that the binding of the compounds (nHAP + 5% SnCl_2_) has generated reactivity between the particles, i.e., a chemical reaction between the particles, forming molecular compounds that are more resistant to erosion/abrasion, in addition to promoting greater sealing of the tubules.

The development of a treatment that increases the acid resistance of dentin in the long term remains a target to be studied. Therefore, more research is needed to achieve optimal protocols for treatments that provide longevity and so that these procedures can be applied clinically, bringing benefits to patients.

Initial curvature analysis to eliminate discrepancies between samples was not performed, which creates a limitation in this study. Future studies are encouraged to use this analysis during sample selection to prevent possible biases in the results. In addition, a long-term evaluation should be performed to assess the effects of the different proposed treatments.

We can conclude that treatment with 5% SnCl2 associated or not with nHAP is a possible alternative in the prevention of erosive-abrasive dentin wear. Thus, clinical studies need to be conducted to clinically prove that 5% SnCl2 associated or not with nHAP can be used in desensitization protocols since it shows excellent obliterating action when analyzed in vitro.
